# Epidemiology of exposure to HIV/AIDS risky conditions in healthcare settings: the case of health facilities in Gondar City, North West Ethiopia

**DOI:** 10.1186/1471-2458-14-1283

**Published:** 2014-12-16

**Authors:** Getahun Kebede Beyera, Teresa Kisi Beyen

**Affiliations:** Department of Environmental and Occupational Health and Safety, Institute of Public Health, College of Medicine and Health Sciences, University of Gondar, Gondar, Ethiopia; Department of Epidemiology and Biostatistics, Institute of Public Health, College of Medicine and Health Sciences, University of Gondar, Gondar, Ethiopia

**Keywords:** HIV/AIDS, Risky conditions, Healthcare workers, Healthcare settings

## Abstract

**Background:**

It has been estimated that every year more than quarter a million health care workers exposed to HIV risky conditions in health care settings, more so in developing countries, with high incidence of HIV/AIDS and unsafe practices. Particularly, Sub-Saharan African countries share at least half of these occupational exposures to HIV risky conditions among health care workers. The aim of this study was to determine the epidemiology of health care workers’ exposure to HIV/AIDS risky conditions and associated factors in the healthcare settings in Gondar city.

**Methods:**

Institution based quantitative cross sectional study was conducted from April 1–20, 2014. The study included 401 health care workers who were selected from the source population by simple random sampling technique. Data were collected by interviewing health care workers using structured and pretested questionnaire. After the collected data entered to EPI INFO version 3.5.3 statistical software and exported to SPSS version 20.0 for analysis, both binary and multivariable logistic regressions were done to identify factors associated with exposure to HIV/AIDS risky conditions.

**Results:**

From a total of 401 health care workers involved in this study, 162(40.4%) reported at least one history of occupational exposure to HIV/AIDS risky conditions in the last one year. More than half (52.31%) of physicians and 47.62% of anesthetists were exposed to HIV/AIDS risky conditions within one year. Lack of training on infection prevention, 5–10 years work experience, long working hours per week, absence of work guidelines, and dissatisfaction with current job were significantly associated with accidental occupational exposure to HIV/AIDS risky conditions.

**Conclusion:**

This study found quite high prevalence of health care workers exposure to HIV/AIDS risky conditions in the health care settings in Gondar city. Therefore, effective and goal oriented educational programmes targeting at health care workers and establishment of surveillance systems for registering, reporting and management of occupational exposures in health care settings are quite important.

## Background

Health care workers (HCWs) are at risk of many infections in health setups [1], such as exposures to human blood and body fluids, placing them at risk for numerous blood-borne infections including human immunodeficiency virus (HIV) [2]. Exposures occur through percutaneous injury (needle-stick or other sharps injury), mucocutaneous injury (splash of blood or other body fluids into the eyes, nose or mouth) or blood contact with non-intact skin [3, 4]. The important factors that influence the overall risk for occupational exposures to blood borne pathogens include the number of infected individuals in the patient population and the type and number of blood contacts [4]. HCWs in areas such as operating, delivery and emergency rooms and laboratories have a higher risk of exposure. Cleaners, waste collectors and others whose duties involve handling blood contaminated items are also at risk [3].

Each day thousands of HCWs, around the world, experience accidental occupational exposures during the course of their role of caring for patients [5]. These exposures can result in a variety of serious and distressing consequences ranging from tremendous anxiety to chronic illness and premature death for the individual involved that can have a negative impact not only on the HCWs, but also their families and colleagues [5, 6].

Of 35 million HCWs worldwide, it has been estimated that every year more than quarter a million health care workers exposed to HIV risky conditions in health care settings. As a result, 1,000 are likely to be infected with HIV [3, 7]. Most of these exposures and infections occur in developing countries, where the prevalence of blood borne pathogens in the general population is high and access to safety devices and protective equipment is limited. However, relatively a small proportion of cases from these regions are documented because systematic surveillance is difficult to undertake and maintain in such environments [8]. It is estimated that 4.4% (range 0.8% to 18.5%) of all HIV infections among HCWs are due to occupational exposures; and at least half of these cases occur in sub-Saharan Africa. Even, these estimates were calculated using the most conservative data available which was generally collected when there was minimal interaction between healthcare systems and patients with HIV/AIDS [5].

While there is still lack of hard data, anecdotal evidence suggests that, in Africa, the health systems may lose one-fifth of their employees due to HIV/AIDS over the next several years [9]. Given the pivotal role of frontline HCWs in resource constrained countries, the potential loss of this number of workers each year is a serious problem that needs urgent attention [5].

In Ethiopia, the information concerning the events surrounding this accidental occupational exposure to HIV/AIDS risky conditions in the health setups is critically lacking. Particularly, there is no published study showing the clear picture of occupational exposure to HIV/AIDS risky conditions among HCWs. Therefore, this study was undertaken to primarily find out the epidemiology and characteristics of health care workers exposure to HIV/AIDS risky conditions in the health care settings in Gondar city, which can then be used as a baseline for developing a surveillance system, preventive guidelines and educational programs.

## Methods

### Study design and area

Institution based quantitative cross-sectional study was conducted to determine epidemiology of health care workers’ exposure to HIV/AIDS risky conditions and associated factors in the healthcare settings in Gondar city, North West Ethiopia.

### Sample size and sampling procedure

Sample size was determined using the formula for single population proportion by considering e 50% prevalence of exposure to HIV/AIDS risky conditions (because there is no previous study), 95% level of confidence and 5% margin of error. Therefore, sample size was determined as follows:


By adding 10% non response rate, the final sample size was 422 HCWs.

In Gondar city, there were a total of 4 public health institutions, 1 hospital and 3 health centers, with a total number of 1032 HCWs. These HCWs were working in outpatient department (OPD), pediatric, maternity, gynecology, medical, surgical, and orthopedic wards, and laboratory, operation, dental, ophthalmology, injection and dressing rooms. The sampling frame consisting of all HCWs in each health institution were obtained from each health institution management. Then, new sampling frame was prepared through compilation of the list of each HCW into a single sampling frame and simple random sampling technique was administered to identify the study subjects.

### Data collection procedure

Data were collected through interviewing health care workers using structured and pre-tested questionnaire.

### Data processing and analysis

Data were entered using EPI INFO version 3.5.3 statistical software and then exported to and analyzed using SPSS version 20.0. Descriptive statistics such as percentages, means and standard deviations were done for most variables in the study. Multivariable logistic regression analysis was done for controlling the possible effects of confounders after bivariable logistic regression analysis has been done by defining exposure to HIV/AIDS risky conditions as a percutaneous injury (needle stick or a cut by sharp objects) or the contact of mucus membranes or non-intact skin with blood, tissue or other body fluids that are considered to be potentially infectious. Finally, variables which have significant association with exposure to HIV/AIDS risky conditions were identified based on AOR with 95% CI and P-value ≤ 0.05.

### Ethical considerations

The study was carried out after getting permission from the ethical review board (IRB) of university of Gondar. Informed consent was also obtained from each health institutions and study participants to participate in the study and confidentiality was granted for information collected from each health institution and study participants by omitting their names from the questionnaires.

## Results

### Socio-demo graphic characteristics

Out of 422 selected HCWs, 401 participated in this study giving 95% response rate. Of the total study participants, 224 (55.9%) were males. Respondents’ age range from 20–60 years with a mean (standard deviation) age of 29.5 (6.75) years. Majority, 249 (62.1%) of the study participants were found in the age group of 20–29 years (Table 1).

**Table 1:** **Socio demographic characteristics of health care workers in Gondar city, North West Ethiopia (n = 401)**

Variables	Number	Percent
Sex		
Male	224	55.9
Female	177	44.1
Age Group		
20-29	249	62.1
30-39	114	28.4
≥ 40	38	9.5
Religion		
Orthodox	316	78.8
Muslim	41	10.2
Protestant	38	9.5
Catholic	6	1.5
Educational level		
Illiterate	3	0.7
Read and write	13	3.2
Primary school	14	3.5
Secondary school	12	3.0
Technical and vocational school	12	3.0
Diploma	62	15.5
Degree and above	285	71.1
Marital status		
Married	185	46.2
Single	194	48.4
Divorced	12	3.0
Widowed	5	1.2
Separated	5	1.2
Job category		
Cleaner	66	16.5
Nurse	142	35.5
Midwifery	49	12.2
Health officer	25	6.2
Laboratory technologist	33	8.2
Anesthetist	21	5.2
Physician	65	16.2
Work experience in years		
<5	285	71.1
5–10	53	13.2
>10	63	15.7

### Epidemiology and characteristics of accidental exposure to HIV/AIDS risky conditions

**Figure 1:**
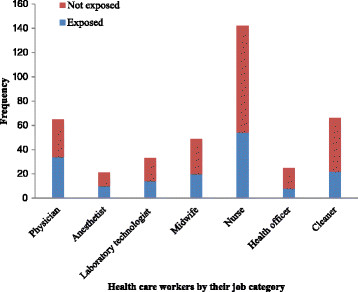
**Exposure status of HCWs to HIV/AIDS risky conditions by their job category in Gondar city, North West Ethiopia (n = 401).** Y-axis represents number of healthcare workers. X-axis represents healthcare workers by their job category. Blue colors- represent number of healthcare workers exposed to HIV/AIDS risky conditions in the last one year. Red colors- represent number of healthcare workers who were not exposed to HIV/AIDS risky conditions in the last one year.

One hundred sixty two (40.4%) HCWs involved in this study reported at least one history of accidental exposure to HIV/AIDS risky conditions in the last one year. Most, 102 (62.96%) of the exposures were mentioned by males and the rest 60 (37.04%) by females. About 47% of the exposed HCWs experienced the exposure more than once, while the rest 53% HCWs exposed just once. Majority, 92 (56.79%) of the exposure episodes occurred through percutaneous injury (needle stick or cut by sharp objects), followed by 90 (55.56%) through contact of mucus membranes and non-intact skin with blood, and 37 (22.85%) through contact of mucus membranes and non-intact skin with tissue or other body fluids that are considered to be potentially infectious. The leading perceived cause of exposure was heavy work load followed by failure to use of protective equipments, and lack of knowledge on standard precautions as reported by 103 (63.58%), 40 (24.69%), 19 (11.73%), respectively. More than half (52.31%) of physicians and 47.62% of anesthetists were exposed to HIV/AIDS risky conditions in the last one year (Figure 1).

Among the exposed HCWs, only 54 (33.33%) of them reported the incidence, while the rest 108 (66.67%) HCWs failed to so. The most common reasons stated by HCWs for not reporting their exposures were: busy work/workload 35 (32.40%), fear of stigma/discrimination 25 (23.15%), lack of support by management 18 (16.67%), reporting is too time consuming 18 (16.67%), and lack of awareness about reporting procedure 12 (11.11%).

### Characteristics in the health institutions

A considerable proportion, 163 (40.6%) of respondents reported that their work place had no safety instructions. Similarly, 124 (30.9%) of the respondents reported that they had no work guideline at their work place. Only 237 (59.1%) of the study participants took training on infection prevention. Majority, 262(65.3%) of the respondents reported that there was not a written protocol for reporting exposure to HIV/AIDS risky conditions at their workplace. More than half (51.1%) of HCWs had worked greater than 48 hours per week (Table 2).

**Table 2:** **Characteristics in the health institutions in Gondar city, North West Ethiopia (n = 401)**

Variables	Number	Percent
Existence of safety instructions at workplace		
Yes	238	59.4
No	163	40.6
Existence of work/procedural guidelines at work place		
Yes	277	69.1
No	124	30.9
Took training on infection prevention		
Yes	237	59.1
No	164	40.9
Existence of written protocol for reporting exposure to HIV/AIDS risk conditions at workplace		
Yes	139	34.7
No	262	65.3
Average number of hours worked per week		
≤ 48	205	51.1
> 48	196	48.9
Availability of safety box at work place		
Yes	396	98.8
No	5	1.2

### Behavioral characteristics of health care workers

Currently, only 29.4%, 6.5%, and 3.5% of the HCWs drink alcohol, chew khat and smoke cigarette, respectively. Despite majority (74.3%) of the HCWs perceived that exposure to HIV/AIDS risky conditions is highly risky, only 42.3% of them never recapped needles at all. Concerning job satisfaction, only 223 (55.6%) HCWs satisfied with their current job (Table 3).

**Table 3:** **Behavioural characteristics of health care workers in Gondar city, North West Ethiopia (n = 401)**

Variables	Number	Percent
Drink alcohol		
Yes	118	29.4
No	283	70.6
Chew chat		
Yes	26	6.5
No	375	93.5
Smoke cigarette		
Yes	14	3.5
No	387	96.5
The risk of exposure to HIV/AIDS risky conditions is		
High	10	2.5
Moderate	17	4.2
Low	76	19.0
Not risky	298	74.3
Recap needles after use		
Never recap	170	42.3
Sometimes	74	18.5
Most of the time	74	18.5
Always	83	20.7
Recap needles after use with		
Single hand	110	47.62
Two hands	121	52.38
Satisfied with current job		
Yes	223	55.6
No	178	44.4
Reason for not satisfied with current job (multiple response is possible)		
Imbalance of salary with work	117	65.73
Poor management	74	41.57
Heavy workload	69	38.76
Exposure to different health hazards	58	32.58
Lack of opportunity for promotion	28	15.73
Lack of cooperation between staffs	8	4.49

### Factors associated with exposure to HIV/AIDS risky conditions

In Bivariable logistic regression analysis, factors such as male gender, physician job category, lack of training on infection prevention, long working hours per week, absence of safety instructions at workplace, recapping needles after use, absence of work guidelines, and dissatisfaction with current job were found to be significantly associated with exposure to HIV/AIDS risky conditions. However, in multivariable logistic regression analysis, lack of training on infection prevention, long working hours per week, 5–10 years work experience, absence of work guidelines, and dissatisfaction with current job became the only independent predictors of accidental exposure to HIV/AIDS risky conditions (Table 4).

**Table 4:** **Determinant factors for exposure to HIV/AIDS risky conditions among health care workers in Gondar city, North West Ethiopia (n = 401)**

		Exposed to HIV/AIDS risky conditions		Crude OR (95% CI)	Adjusted OR (95% CI)
Variables		Yes	No		
Sex	Male	102	122	1.63(1.09, 2.45)*	1.84(0.98, 3.48)
	Female	60	117	1.00	
Job category	Cleane	22	44	1.00	
	Nurse	54	88	1.23(0.66, 2.27)	
	Midwifery	20	29	1.38 (0.64, 2.97)	
	Health officer	8	17	0.94(0.35, 2.52)	
	Laboratory technologist	14	19	1.47(0.62, 3.48)	
	Anesthetist	10	11	1.82 (0.67, 4.93)	
	Physician	34	31	2.19(1.08, 4.45)*	
Work experience years	<5	116	169	1.00	
	5-10	24	29	1.21(0.67, 2.18)	2.81(1.15, 6.86)*
	>10	22	41	0.78(0.44, 1.38)	1.27(0.49, 3.34)
Took training on infection prevention	Yes	40	197	1.00	
	No	122	42	14.31(8.78, 23.31)*	4.49(2.27, 8.89)*
Average number of hours worked per week	≤48	21	184	1.00	
	>48	141	55	22.46(12.98, 38.88)*	9.8(5.13, 18.74)*
Existence of safety instructions at workplace	Yes	69	169	1.00	
	No	93	70	3.25(2.14, 4.94)*	
Existence of work guidelines at workplace	Yes	77	200	1.00	
	No	85	39	5.66(3.57, 8.98)*	2.06(1.03, 4.1)*
Recap needles after use	Never recap	52	118	1.00	
	Sometimes	36	38	2.15(1.23, 3.77)*	
	Most of the time	38	36	2.40(1.37, 4.20)*	
	Always	36	47	1.74(1.01, 2.99)*	
Satisfied with current job	Yes	37	186	1.00	
	No	125	53	11.86(7.36, 19.11)*	6.62(3.53, 12.43)*

*Significant at p <0.05.

## Discussion

This study highlighted HCWs accidental exposure to HIV/AIDS risky conditions and predicting factors in the health care settings in Gondar city, Ethiopia. Almost in agreement with the study done in Iran, where 43.4% HCWs had at least one occupational exposure to blood and other infected fluids [10], 40.4% HCWs in this study reported occupational exposure to HIV/AIDS risky conditions. However, this figure is greater than the finding of the study conducted in Nepal, where only 28% of HCWs exposed to HIV risky conditions [11]. The difference might be due to difference in setting, work environment and available resources. Inadequate staff, insufficient training, duty overload and fatigue may lead to occupational exposures [12].

Consistent with other studies [13–15], the most common mode of exposures to HIV/AIDS risky conditions was through needle prick/cut by sharps. This could be attributed to the frequent use of sharp objects, especially needles, as many procedures (e.g. drawing blood, accessing a vein to start intravascular fluids administration) require their use [16]. Even though health care workers have repeated a given procedure hundreds of time, one slip can cause injury with potentially serious consequences; an unexpected or sudden movement by the patient, work colleague or a momentary lack of concentration can result in injury [17]. Other possible reasons for high prevalence of sharp injuries include lack of specific programme measures to address occupational challenges such as failure to use personal protective equipments (PPEs), lack of safer sharp devices, lack of information and non adherence to standard precautions [18].

Compared to other HCWs, higher proportion (52.31%) of physicians exposed to HIV/AIDS risky conditions. This result is comparable with the study done in Tanzania [19], where doctors and nurses had high prevalence of occupational exposures than other HCWs. This could be explained by the fact that physicians may perform more risky procedures overall or have a higher number of patients as compared to other HCWs since proportion of physicians is very low in Ethiopian scenario. This considerably higher rate of exposure in this category of HCWs indicates that they are the priority areas for infection control in the health facilities.

Although occupational exposure to HIV/AIDS risky conditions should be reported, in this study, a large proportion of HCWs (66.67%) failed to do so. This figure is higher compared to the studies done in Kenya [18] and Tanzania [19], where 45% and 33.33% HCWs did not report their exposures, respectively. Not reporting accidental occupational exposure to HIV/AIDS risky conditions increases the risk of HIV infection, since no post exposure preventive measures were taken to reduce the risk of infection. Moreover, underreporting may be related to unwillingness to reveal incidence or lack of motivation due to the belief that HCWs can handle the issue themselves.

In this study, the major reasons articulated by HCWs for not reporting their exposures are in agreement with previous studies [20–22]. HCWs tend to blame busy work as a reason for not reporting their exposures, but even later they forget it do so [23].

Consistent with previous studies [18, 24], lack of training on infection prevention significantly elevated the odds of exposure to HIV/AIDS risky conditions. This could be explained by the fact that training enhances awareness and improves skills among HCWs. HCWs within 5–10 years work experience were more likely to experience exposure to HIV/AIDS risky conditions compared to those who had less than 5 years work experience. This might be attributed to the fact that most of the HCWs who had less than 5 years work experience took training on infection prevention, which enhances their skills and knowledge pertaining to health and safety precautions. But, in other study the prevalence of occupational exposure was high among those with experience less than 10 years [18].

It is recognized that adverse schedule characteristics such as long work hours significantly increased the risk of occupational exposure [25, 26]. In line with this, the current study depicted that working more than 48 hours per week significantly increased history of reporting occupational exposure to HIV/AIDS risky conditions in one year compared to those who had worked less or equal to 48 hours per week. This could be justified by the fact that working excessive hours can result in stress, emotional and physical exhaustion, which are likely to increase the chance of human error and contribute to a tendency towards risky behaviors and poor compliance with the precautions in general [27].

Existence of work/procedural guideline at workplace was also associated with occupational exposure to HIV/AIDS risky conditions. Those HCWs who lack work guidelines at their workplace were more likely report one year history of occupational exposure to HIV/AIDS risky conditions compared to their counter parts. This might be linked to the fact that those HCWs who lack work guideline may fail to follow the correct procedures, such as safe injections while they play their role of caring patients in such a high risky environment.

Consistent with previous study [27], HCWs who dissatisfied with their current job were more likely report exposure to HIV/AIDS risky conditions than those who satisfied with their current job. Probably carelessness, stress and emotional upset which could results in poor compliance with health and safety issues for prevention of occupational exposures are incidents accompany dissatisfaction.

## Conclusion

This study found high prevalence of health care workers’ exposure to HIV/AIDS risky conditions in the healthcare settings in Gondar city. This fact puts HCWs into a great risk for acquiring health care associated HIV infection. Lack of training on infection prevention, lack of work guidelines at workplace, long working hours, and dissatisfaction with current job were avoidable factors which raised the likelihood of HCWs exposure to HIV/AIDS risky conditions. Therefore, effective and goal oriented educational programmes targeting at HCWs and establishment of surveillance systems for registering, reporting and management of occupational exposures in health institutions are required. Specifically, hospitals and health centers should provide training on infection prevention, safety guidelines, and should reduce long working hours. The use of protective measures and post exposure prophylaxis (PEP) utilization against accidental occupational exposure to HIV/AIDS risky conditions are also important ways to prevent viral transmission among HCWs. Moreover, strengthening health systems and highlighting occupational exposure is necessary to prevent further spread of blood borne pathogens including the current Ebola outbreak in general, not just HIV/AIDS.
